# Fighting the COVID-19 pandemic in Ghana: a report from the Kwame Nkrumah University of Science and Technology, Kumasi

**DOI:** 10.11604/pamj.supp.2020.37.1.25749

**Published:** 2020-12-09

**Authors:** Nana Kwame Ayisi-Boateng, Arti Singh, Joseph Abu-Sakyi, Phyllis Tawiah, Ivy Darkwa, Osei Kwaku Wusu-Ansah

**Affiliations:** 1 Department of Medicine, Kwame Nkrumah University of Science and Technology, Kumasi, Ghana,; 2 University Hospital, Kwame Nkrumah University of Science and Technology, Kumasi, Ghana,; 3 School of Public Health, Kwame Nkrumah University of Science and Technology, Kumasi, Ghana

**Keywords:** COVID-19, university hospital, Kumasi, Ghana

## Abstract

The corona virus disease 2019 (COVID-19) has stretched the resources of health facilities but there is a lack of context-specific reports across Africa. Since February, 2020, the university hospital, Kwame Nkrumah University of Science and Technology (KNUST), a district-level institution, has been at the forefront in contributing to efforts in Ghana to fight the global pandemic. As of 16^th^ August, 2020, 1755 individual samples have been taken at the hospital for COVID-19 out of which 629 (35.8%) tested positive, 414 (65.9%) recoveries and 6 (0.95%) deaths. The hospital's out-patient attendance has reduced by almost 50% with attendant loss of revenue. Here in, we present a report on our activities, highlight lessons and recommendations that other health facilities can glean from.

## Commentary

**Figure 1:**
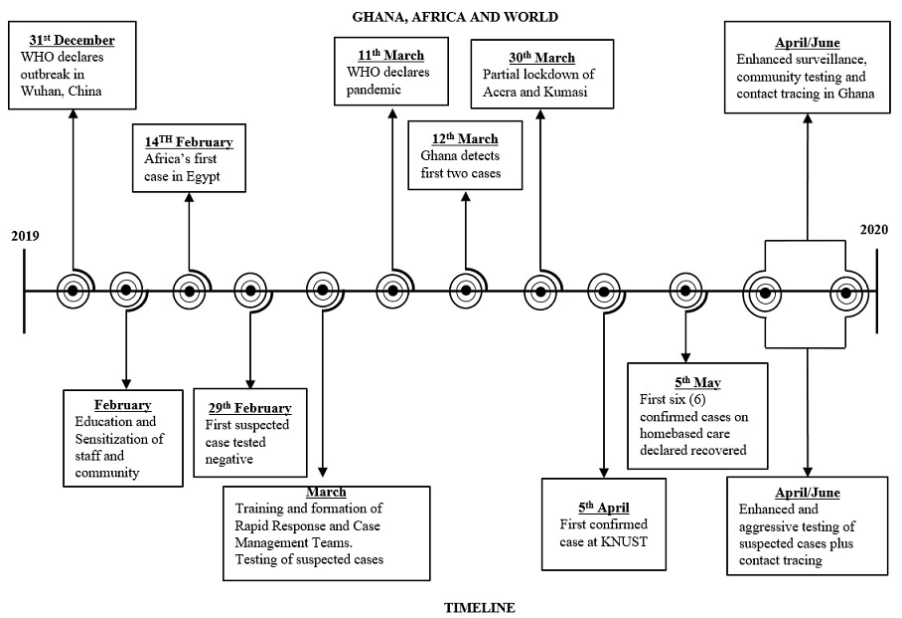
global, national and university hospital timelines

On 31^st^ December, 2019, the World Health Organization (WHO) office in Wuhan Province, China, reported an outbreak of severe acute respiratory syndrome coronavirus-2 (SARS-CoV-2) with associated coronavirus disease (COVID-19). Since then, significant events have occurred (Figure 1) and the disease has posed an unprecedented threat to both public health and the global economy. Ghana has been reputed as one of the most aggressive countries in testing for COVID-19 in Africa. As of 16^th^ August, 2020, a total of 428,695 tests using real time polymerase chain reaction (RT-PCR) have been conducted with a test positivity rate of 10.0%. Out of 42,993 confirmed cases, 40,796 (94.9%) have recovered and 248 (0.58%) have died [1]. Ghana, like several other African countries, is faced with a myriad of health system challenges. Health facilities are already overstretched with the routine service needs and are at risk of amplifying the COVID-19 scourge. In this light, we provide an overview of the main activities (between February and August, 2020) in handling the COVID-19 pandemic at our hospital. We also highlight the main challenges and recommendations to provide decision-makers with actionable information on healthcare service preparedness.

**Setting:** the university hospital, located within the Kwame Nkrumah University of Science and Technology (KNUST), is a district level (primary care) hospital in Kumasi in the Ashanti Region. It is a 125-bed quasi-government facility, which is equipped to provide general and specialist services to university staff and their dependents, students and private patients. It has a catchment population of approximately 300,000.

**Staff training, public education and outbreak preparedness:** seven rapid response teams (on duty for each day of the week) were formed, building from existing response teams during the 2014 Ebola Virus Disease (EVD) threat in Ghana [2] and the 2017 and 2019 outbreak of Influenza A (H1N1 and H3N2) within the Kumasi Metropolis [3]. The team comprised a cadre of doctors, nurses, laboratory staff, pharmacists, drivers, cleaners and security. To equip staff of the hospital to deal with the threat of COVID-19 outbreak, a series of training workshops were organized in the hospital. In all, approximately 300 staff drawn from various units and sections participated in the workshops. The training focused on the epidemiology, clinical presentation, infection prevention, and case management, appropriate use of personal protective equipments (PPEs) and sample taking. Information, education and communication (IEC) materials were prepared and disseminated for public education on prevention of infection spread, symptoms of COVID-19 as well as types of facemasks and their effective uses. Several media presentations were held on public education and preventive strategies.

**Case detection, case management and contact tracing:** the processes of case detection, case management and contact tracing were based on guidelines by the Ghana Health Service (GHS) and the Ministry of Health [4]. Using the case definition of COVID-19, the hospital employed a screening checklist at the pre-triage area to enable early identification of suspected cases before they entered the hospital. A suspected case was isolated, moved to the holding area and appropriate samples taken for testing. Samples included naso- and oropharyngeal swabs, nasopharyngeal wash and sputum and RT-PCR tests were conducted at the Kumasi Centre for Collaborative Research into Tropical Medicine (KCCR) situated on KNUST campus. Our rapid response and case management teams partner the Ashanti Regional Health and the Oforikrom Municipal Health Directorates in following up confirmed cases. Confirmed cases who were asymptomatic or had mild symptoms underwent home-based management or were admitted to the Regional Isolation centres. The university hospital has a 6-bed treatment centre that admits mild to moderately ill patients. Those with clinical presentations categorized as severe or critical are referred to the Komfo Anokye Teaching Hospital (KATH), a tertiary institution within the Kumasi Metropolis. The Public Health Unit of the hospital teams up with community-based disease control officers to undertake contact tracing for all confirmed cases. We also engage the services of the KNUST counselling Unit to provide psychological support to both suspected and confirmed cases and their families.

**Situation report and analysis:** from 5^th^ April, 2020 when the first case of COVID-19 was confirmed at the university hospital, the number of new cases has increased exponentially. As of 16^th^ August, 2020, 1755 samples have been taken out of which 629 tested positive for COVID-19 (positivity rate 35.8%). Approximately 52% (327) of the positive cases were males and the age group with the highest frequency was 20-29. The youngest was 17 days and the oldest was 89 years. Majority (66.6%; n = 419) of confirmed cases had mild symptoms and 201 (32.0%) were classified as asymptomatic. We recorded 6 mortalities (case fatality ratio 0.95%) (Table 1) which involved patients with severe underlying comorbidities such as stroke, heart failure, hypertension and diabetes.

**Table 1:** summary of institutional, national and global data as of 16^th^ August, 2020

Indicators	KNUST	National (Ghana)	Global
**Total number of confirmed cases**	629	42,993	21,294,845
**Number of deaths**	6	248	761,779
**Case fatality rate (%)**	0.95	0.58	3.6

**Challenges and lessons learnt:** at the end of 2019, the total out-patient attendance at the university hospital was 136,769 with an average monthly attendance of 11,397. In 2020, patient attendance for January and February was 14,083 and 16,872 respectively. However, since 12^th^ March, 2020 when COVID-19 cases were detected in Ghana, imposition of partial lockdown on 30^th^ March, and up to the end of June, patient attendance has plummeted to between 5,000 and 7,000 patients (almost 50% drop) with an attendant reduction in hospital revenue. A significant proportion of the hospital's budget has also been expended on the purchase of PPEs and other COVID-19-related activities. Increasing health workers' exposure and infection rate result in untold stress and staff deficiency. Samples of suspected cases taken at the university hospital are sent to KCCR, the only accredited centre for COVID-19 testing in the Ashanti region and northern sector of the country. Initially, we received test results within 4 to 6 hours after submission. We were able to keep suspected cases in our holding area (capacity of 6 patients) until results were obtained. As the number of cases escalated and the demand on KCCR increased, test results were delayed for between 48 to 72 hours, sometimes with a backlog up to 7 days. This made it impossible to hold suspected cases, especially asymptomatic or those with mild symptoms. Majority of suspected cases whose samples were taken were given face masks and counselled to self-isolate at home. It was disappointing to find that by the time their results were ready and were contacted, some of those patients had flouted the self-isolation policy. A number of them disputed positive test results since they were purportedly well. Some of them provided false addresses or their phone numbers could not be reached. Poor road networks as well as lack of proper address system posed a great challenge to home-based care and contact tracing teams. Confirmed cases who were managed at home faced stigmatisation from neighbours as hospital-branded vehicles and PPE-wearing health workers paid visits to them. However, because Ashanti region has only two isolation centres with a total of 156 rooms, 89.6% (362) of confirmed cases so far from our hospital have been managed at home.

In conclusion, the Government of Ghana, Ministry of Health and its agencies have demonstrated commitment and commendable efforts in the global fight against the COVID-19 pandemic. The significant impact of COVID-19 on OPD attendance and revenue notwithstanding, the university hospital, KNUST, is proud to have contributed to these efforts. We believe our experience and challenges mirror stories in other African countries. We therefore recommend a concerted effort by African policy makers, health administrators and researchers to develop context-specific solutions to minimise the deleterious effects of COVID-19.
